# Correction of nonuniformity error of Gafchromic EBT2 and EBT3

**DOI:** 10.1120/jacmp.v17i3.5862

**Published:** 2016-05-08

**Authors:** Toshizo Katsuda, Rumi Gotanda, Tatsuhiro Gotanda, Takuya Akagawa, Nobuyoshi Tanki, Tadao Kuwano, Kouichi Yabunaka

**Affiliations:** ^1^ Faculty of Human Relation Tokai Gakuin University Kakamigahara‐city Gifu Japan; ^2^ Department of Radiological Sciences Ibaraki Prefectural University of Health Sciences Ishiki Ibaraki Japan; ^3^ Faculty of Health Sciences Junshin Gakuen University Minami‐ku Fukuoka Japan; ^4^ Department of Radiological Technology Tokushima Red Cross Hospital Komatsujshima Tokushima Japan; ^5^ RIKEN Center for Life Science Technologies Cyuou‐ku, Kobe‐city Hyogo Japan; ^6^ Graduate School of Health Sciences, Okayama University Okayama‐city Okayama Japan; ^7^ Graduate School of Medicine, University of Tokyo Bunkyou‐Ku Tokyo Japan

**Keywords:** computed tomography, absorbed dose, Gafchromic film, nonuniformity error, ultraviolet

## Abstract

This study investigates an X‐ray dose measurement method for computed tomography using Gafchromic films. Nonuniformity of the active layer is a major problem in Gafchromic films. In radiotherapy, nonuniformity error is reduced by applying the double‐exposure technique, but this is impractical in diagnostic radiology because of the heel effect. Therefore, we propose replacing the X‐rays in the double‐exposure technique with ultraviolet (UV)‐A irradiation of Gafchromic EBT2 and EBT3. To improve the reproducibility of the scan position, Gafchromic EBT2 and EBT3 films were attached to a 3‐mm‐thick acrylic plate. The samples were then irradiated with a 10 W UV‐A fluorescent lamp placed at a distance of 72 cm for 30, 60, and 90 minutes. The profile curves were evaluated along the long and short axes of the film center, and the standard deviations of the pixel values were calculated over large areas of the films. Paired *t*‐test was performed. UV‐A irradiation exerted a significant effect on Gafchromic EBT2 (paired *t*‐test; p=0.0275) but not on EBT3 (paired *t*‐test; p=0.2785). Similarly, the homogeneity was improved in Gafchromic EBT2 but not in EBT3. Therefore, the double‐exposure technique under UV‐A irradiation is suitable only for EBT2 films.

PACS number(s): 87.53 Bn

## I. INTRODUCTION

Gafchromic films are used for measuring X‐ray doses of diagnostic examination, and the half‐value layer and effective energy are indicators of quality assurance and control.^1^, ^2^, ^3^, ^4^, ^5^ Gafchromic films are also applicable to X‐ray dose measurements in computed tomography (CT).^6^, ^7^, ^8^, ^9^, ^10^, ^11^, ^12^

In the CT dose measurements, a Gafchromic film is rolled up with a certain thickness of flexible acrylic sheet,^(7)^ enabling three‐dimensional X‐ray dose measurements in CT.^7^, ^8^ Several pieces of Gafchromic films are placed between the flexible acrylic sheets, whose thickness determines the spatial resolution of the dose measurement. Nonuniformity error is a critical problem in dose measurements using Gafchromic films.

Alternatively, Gafchromic films can be shaped into hemicylindrical acrylic phantoms. In this method, the Gafchromic film is sandwiched between the phantoms and is scanned during CT, enabling radiation dose measurements of single or helical scans and conical‐beam CT. This technique achieves a virtual three‐dimensional rendering of the dose distribution.^(12)^

The active layers of Gafchromic EBT2 and EBT3 contain a yellow dye. Variations in the color density of this dye indicate irregularities in the active layer of the film. The information on radiation dose and active layer thickness is obtained as image information on the red and blue channels, respectively. Such information can be exploited to correct the nonuniformity error.^13^, ^14^ In addition, by combining the X‐ray double‐exposure technique before true irradiation with the red‐channel information, scans increase once; however, the nonuniformity reduction is not improved from that of multichannel analysis.^(15)^ The X‐rays of the double‐exposure technique can be replaced by density increases in Gafchromic films irradiated with ultraviolet (UV) rays.^(16)^

Because the active layer of a Gafchromic film reacts to UV rays,^17^, ^18^ UV irradiation is effective only if appropriately applied. For instance, UV irradiation can markedly improve the uniformity of Gafchromic EBT.^(16)^ However, the effect of UV irradiation on Gafchromic EBT2 and EBT3 has not been considered, although Gafchromic EBT2 and EBT3 are known to effectively react to UV‐A.^(19)^

This study aims to correct the nonuniformity error in Gafchromic EBT2 and EBT3 by irradiating them with UV‐A, as an X‐ray replacement in the double‐exposure technique.

## II. MATERIALS AND METHODS

### A. Gafchromic film EBT2 and EBT3

The Gafchromic films used in this study were Gafchromic EBT2 (Lot# 02171403; expiry date February 2016) and Gafchromic EBT3 (Lot# 04011401; expiry date April 2016), both purchased from Ashland Inc. (Covington, KY). The Gafchromic EBT2 and EBT3 samples were sized 20 cm×25 cm, and their dosimetry ranged from 1 to 800 cGy. A Gafchromic film is a self‐developing film that requires no developing chemicals or processes. The red‐channel data were used in the image analysis.^(14)^ Although both EBT2 and EBT3 are transmission‐type films, scanning was performed in the reflection mode because the nonuniformity error is small in this mode.^(20)^

### B. Gafchromic EBT size

The short and long axes of the Gafchromic EBT2 and EBT3 samples (20 and 25 cm, respectively) were designed for scanning with a flatbed scanner and uniform irradiation under black light. One piece each of Gafchromic EBT 2 and EBT3 was prepared and marked in the top right corner. The front sides of the films were affixed to a 3‐mm‐thick acrylic board.

### C. UV‐ray irradiation

Since the front side of EBT2 is coated with a UV‐protection layer, the Gafchromic EBT2 and EBT3 samples were irradiated with UV‐A light from their back sides.^(19)^ The UV fluorescent lamp (10 W black light, NEC FL10SBL; NEC Lighting, Ltd. Tokyo, Japan) generated UV‐A rays with a peak wavelength of 365 nm. The Gafchromic EBT 2 or EBT3 sample was positioned 72 cm from the black light surface (Fig. 1). During the experiment, the irradiation area was surrounded with 3‐mm‐thick UV‐ray‐cutting acrylic plates (COMOGRAS CG UV40 P; Lot # 140406C, B Kuraray Co., Ltd., Tokyo, Japan) to protect the experimenters. The UV‐ray strength was measured by a UV meter (UVR‐300; Topcon Technohouse Co., Tokyo, Japan) with a UD=360 probe (365 nm).

The UV‐ray strength at 72 cm was $0.0743\;\text{mW}/{\text{cm}}^{2}$. Gafchromic EBT2 and EBT3 samples were exposed to UV rays for 30, 60, and 90 min (Fig. 2). During the irradiation and image acquisition, the room temperature was maintained at 21°–25°C.

**Figure 1:**
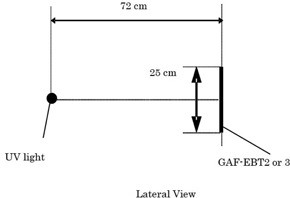
Lateral view of the arrangement of UV‐A irradiation. GAF=Gafchromic film.

**Figure 2:**
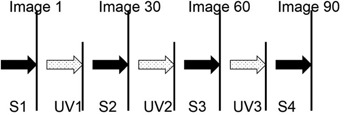
UV‐A irradiation and scan image timing. S1 to S4: image scan, UV1 to UV3: UV‐A irradiation.

### D. Image acquisition

Before and after each UV‐A irradiation, the Gafchromic EBT2 or EBT3 sample was scanned with a flatbed scanner (Epson ES‐10000G; Seiko Epson Co., Nagano, Japan) using Adobe Photoshop CS2 (Adobe Systems Inc., San Jose, CA). Data were read in the RGB mode (48‐bit, 100 dpi resolution) with a PPC film (CR‐PP686; 3M Company, St. Paul, MN). Moiré artifacts (Newton's rings) were removed by applying a protective film of liquid crystal (LCD‐230W; Sanwa Supply Inc., Okayama, Japan).^(4)^ The Gafchromic EBT2 and EBT3 films were always scanned in the landscape direction. The UV‐A irradiation and scanning were carried out with the Gafchromic film fixed to the acrylic plate. The acrylic plate improved the scan position reproducibility of the Gafchromic films (see Fig. 3).

**Figure 3:**
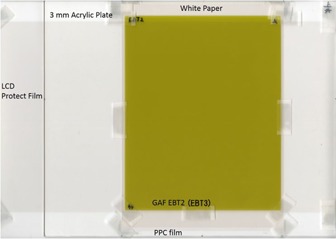
Acrylic plates with Gafchromic EBT3. GAF=Gafchromic film; LCD=liquid crystal display.

### E. Images for analysis

The scanned image data were analyzed by Image J version 1.44o image analysis software for Macintosh (National Institutes of Health, Bethesda, MD). Noise caused by dust and scratches was removed by median filter preprocessing with a 2‐pixel radius.^(21)^ Images were split into R, G, and B channels and used as 16‐bit R‐channel images. When making a subtraction image, the short‐ and long‐time UV‐A irradiation images were used to correct the nonuniformity error and to substitute the real X‐ray image, respectively. Six subtraction images (three noncorrected and three corrected images) were acquired from each film and analyzed along their short and long axes to obtain the profiles.

### F. Profile plots

Figure 4 shows representative sites of the profile measurements along the long axis (line C) and the short axis (line D) of an image. The region enclosed by A is the Gafchromic film area. To remove the cutting effects, a frame of 40 pixels (approximately 10 mm) from the edge of each image was excluded. The measurement of one pixel line was difficult to precisely locate on all images because the scan position of the Gafchromic EBT2 or EBT3 was slightly rotated on the flatbed scanner. Therefore, the profiles were generated over a small area (10 pixels width) rather than a one‐pixel line.

**Figure 4:**
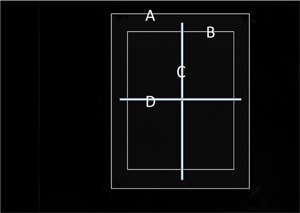
Position of profile curves and ROI for SD. Line A: Gafchromic film area. Line B: ROI for SD area. Line C: long profile curve line. Line D: short profile curve line.

### G. Data analysis

#### G.1 Profiles

The profile curves were plotted along the long and short axes of each corrected and noncorrected subtraction image, and their differences were compared.

#### G.2 Standard deviation (SD)

The region of interest (ROI) of the data acquisition area (20 cm×15 cm) was centered on the Gafchromic film (area B in Fig. 4), and the SD of the pixel values was measured. Paired *t*‐tests were performed by the statistical software JMP ver. 4 (SAS Institute Inc., Cary, NC).

## III. RESULTS

### A. UV‐A strength

The strength of the irradiated UV‐A was $0.0743\;\text{mW}/{\text{cm}}^{2}$. Over 30, 60, and 90 min, the irradiated UV‐A was delivered at 2.23, 4.45, and 6.68 mJ/cm^2^, respectively.

### B. Profile evaluations

#### B.1 Gafchromic EBT2

5, 6 showed the profile curves along the long and short axes of the EBT2 images, respectively. Results of both corrected and noncorrected subtraction images are shown.

**Figure 5:**
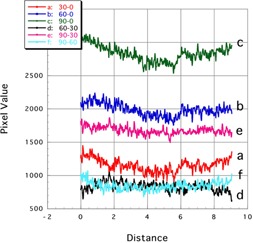
Profile curves (long axis) of subtraction images of Gafchromic EBT2. Lines a: 30‐0, b: 60‐0, and c: 90‐0 showed the noncorrected subtraction image. Lines d: 90‐30, e: 90‐60, and f: 60‐30 showed the corrected subtraction image.

**Figure 6:**
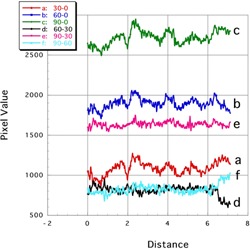
Profile curves (short axis) of subtraction images of Gafchromic EBT2. Lines a: 30‐0, b: 60‐0, and c: 90‐0 showed the noncorrected subtraction image. Lines d: 90‐30, e: 90‐60, and f: 60‐30 showed the corrected subtraction image.

##### B.1.1 Long axis

Along the long axis of the noncorrected subtraction images, the pixel values were lower at the center than at both sides (Fig. 5; profiles a, b, and c). This indicates an insufficient smoothing effect of the yellow dye. However, the nonuniformity error was reduced in the corrected (UV‐A irradiated) images (Fig. 5; profiles d, e, and f). The profiles were almost fat, indicating that the error was sufficiently corrected.

##### B.1.2 Short axis

Along the short axis of the noncorrected subtraction images, the pixel values were decidedly uneven, being high at one side and low at the other (Fig. 6; profiles a, b, and c). Therefore, the yellow dye exerted little effect against nonuniformity. The nonuniformity error was reduced in the corrected subtraction images (Fig. 6; profiles d, e, and f). The flatness of the latter profiles again indicates sufficient correction of the nonuniformity.

#### B.2 Gafchromic EBT3

7, 8 show the profile curves along the long and short axes of the EBT3 film, respectively. Results are shown for both noncorrected and corrected subtraction images.

**Figure 7:**
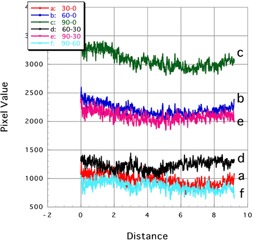
Profile curves (long axis) of subtraction images of Gafchromic EBT3. Lines a: 30‐0, b: 60‐0, and c: 90‐0 showed the noncorrected subtraction image. Lines d: 90‐30, e: 90‐60, and f: 60‐30 showed the corrected subtraction image.

**Figure 8:**
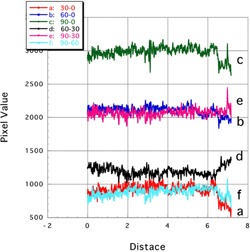
Profile curves (short axis) of subtraction images of Gafchromic EBT3. Lines a: 30‐0, b: 60‐0, and c: 90‐0 showed the noncorrected subtraction image. Lines d: 90‐30, e: 90‐60, and f: 60‐30 showed the corrected subtraction image.

##### B.2.1 Long axis

Along the long axis, the profile curves were almost flat and of similar shape, regardless of whether they were acquired from noncorrected subtraction images (Fig. 7; profiles a, b, and c) or corrected images (Fig. 7; profiles d, e, and f).

##### B.2.2 Short axis

Comparing the profile curves along the short axis in the noncorrected (Fig. 8; profiles a, b, and c) and corrected (Fig. 8; profiles d, e, and f) subtraction images of Gafchromic EBT3, we observe similarly flat profiles in both cases.

### C. Comparison of SDs

#### C.1 EBT2

As shown in Table 1, the SD in the ROI of the noncorrected EBT2 subtraction images was maximized at ±136.14 pixels (in the 90–0 min image) and minimized at±109.72 pixels (in the 30–0 min image). The average SD of the pixel values in the ROIs of these images was ±121.83.

In contrast, in the corrected EBT2 subtraction images, the SD was maximized at ±76.32 pixels (in the 60–30 min image) and minimized at ±70.48 pixels (in the 90‐30 min image). The average SD of the pixel values in the ROIs of these images was ±73.81.

These results show that UV‐A irradiation reduced the SD of the pixel values in images of EBT2 films. The SD in the corrected subtraction images was almost half that in the uncorrected subtraction images. This difference was statistically significant (paired *t*‐test; p=0.0275).

**Table 1:** SD value of noncorrected and corrected subtraction images of EBT2 films.

*Noncorrected Image*				
*Time*	*30–0*	*60–0*	*90–0*	*Average*
SD	±109.72	±119.63	±136.14	±121.83
*Corrected Image*				
*Time*	*30–0*	*60‐0*	*90–0*	*Average*
SD	±76.32	±70.47	±74.65	±73.81

p<0.05

#### C.2 EBT3

Table 2 presents the pixel‐value SDs in the EBT3 films. The SD in the ROIs of noncorrected EBT3 subtraction images was maximized at ±179.65 pixels (in the 90‐0 min image) and minimized at ±128.87 pixels (in the 60‐0 min image). The average SD of the pixel values in the ROIs of these images was ±155.24.

**Table 2:** SD value of noncorrected and corrected subtraction images of EBT3 films.

*Noncorrected Image*				
*Time*	*30–0*	*60–0*	*90–0*	*Average*
SD	±157.20	±128.87	±179.65	±155.24
*Corrected Image*				
*Time*	*30–0*	*60–0*	*90–0*	*Average*
SD	±124.69	±136.29	±130.05	±130.35

p>0.05

In the ROIs of corrected subtraction images, the SD was maximized at ±136.29 pixels (in the 90‐30 min image), minimized at ±124.69 pixels (in the 60‐30 min image), and averaged at ±130.35 pixels.

According to these results, the SDs of the pixel values in the EBT3 subtraction images were not statistically different in the corrected and noncorrected cases (paired *t*‐test; p=0.2785). Therefore, the EBT3 film was not improved by UV‐A irradiation.

## IV. DISCUSSION

### A. Effects of UV‐A irradiation

The profile curves of Gafchromic EBT2 in the corrected subtraction images were flatter than those in the noncorrected subtraction images, indicating that UV‐A irradiation effectively corrected the nonuniformity error in the active layer of this film, relative to red‐channel analysis of the yellow dye alone. However, UV‐A irradiation conferred no benefit on Gafchromic EBT3.

### B. Reproducibility of scan position

When scanning Gafchromic EBT2 or EBT3 with the flatbed scanner, the position of the film tends to drift, degrading the precision of the subtraction images. Thus, subtraction images cannot be simultaneously acquired on a pixel‐by‐pixel basis. For this reason, the Gafchromic EBT2 and EBT3 samples were affixed to 3‐mm‐thick acrylic plates prior to UV‐A irradiation and image scanning. The acrylic plate was considered to improve the consistency of the scan position.

### C. Analysis area

Radiochromic films can be cut with scissors into arbitrarily sized pieces. However, the cutting pressure separates the layers at the film edge. Because the layer separation extends up to 8 mm,^(14)^ a frame of 40 pixels (approximately 1 cm) from the film edges was excluded from the data collection.

#### C.1 Profile curves

As noted above, the scan positions of the film were slightly inconsistent. Therefore, when plotting a profile curve, the selected pixels may not be correctly aligned. To reduce this problem, the profile data were selected from a two‐dimensional area (10 pixels width) and the profile was plotted from the mean value.

#### C.2 SD

While the profile data were evaluated over an extremely small area, the paired *t*‐test of the pixel‐value SDs was conducted over a large area (covering almost the entire ROI).

### D. UV protection for humans

As UV‐A is detrimental to the human body,^22^, ^23^ the experimenter was protected by a UV irradiation box constructed from UV‐ray‐cutting acrylic boards. There was no leakage of UV rays from the box.

### E. Future study

Black light with a peak wavelength of 365 nm increased the density of the active layer in Gafchromic EBT2 and EBT3 and effectively corrected the nonuniformity error in EBT2. In future study, suitable strengths of the UV‐A irradiation should be determined.

## V. CONCLUSIONS

We evaluated a method for correcting nonuniformity error in Gafchromic films. Specifically, we removed the density irregularities caused by uneven thickness of the active layer by irradiating the films with UV‐A rays.

UV‐A irradiation effectively reduced the nonuniformity in Gafchromic EBT2, indicating that this technique can substitute for the X‐ray double‐exposure technique. Therefore, this technique is applicable to diagnostic examinations such as CT.

However, in the EBT3 film, the UV‐A‐irradiated film was not improved over the untreated film containing the yellow dye.

## ACKNOWLEDGMENTS

This study was supported by JSPS KAKENHI Grant Number 26460740.

## COPYRIGHT

This work is licensed under a Creative Commons Attribution 4.0 International License.

## Supporting information

Supplementary Material
