# Supplementary material for: Correction of nonuniformity error of Gafchromic EBT2 and EBT3
Source: J Appl Clin Med Phys. 2016 May 8;17(3):41–51. doi: 10.1120/jacmp.v17i3.5862 (PMC5690916; doi:10.1120/jacmp.v17i3.5862)
Supplement: Supplementary file 1 — Supplementary Material [file ACM2-17-041-s001.docx]

Correction of nonuniformity error of Gafchromic EBT2 and EBT3

**Toshizo Katsuda, Ph. D.**

Faculty of Human Relation, Tokai Gakuin University,

*5-68, Nakakirino-cyo, Kakamigahara-city, Gifu, 504-8511, Japan*

tkatsuda@tokaigakuin-u.ac.jp

(Corresponding author)

Running title: UV exposure of Gafchromic film

**Rumi Gotanda, M. Sc.**

Department of Radiological Sciences, Ibaraki Prefectural University of Health Sciences,

*4669-2, Ami, Ishiki, Ibaraki, 300-0394, Japan*

[gotanda@ipu.ac.jp](mailto:gotanda@ipu.ac.jp)

**Tatsuhiro Gotanda, Ph. D.**

Faculty of Health Sciences, Junshin Gakuen University,

*1-1-1, Chikushigaoka, Minami-ku, Fukuoka, 815-8510, Japan*

[gotanda.t@junshin-u.ac.jp](mailto:gotanda.t@junshin-u.ac.jp)

**Takuya Akagawa, M. Sc.**

Department of Radiological Technology, Tokushima Red Cross Hospital,

*103, Irinokuchi, Komatsujshima, Tokushima, 773-8502, Japan*

th_sl_f_jpn@yahoo.co.jp

**Nobuyoshi Tanki, M. Sc.**

Center for life science technologies, RIKEN

*2-2-3, Minatojimaminami-cyo, cyuou-ku, Kobe-city, Hyogo, 650-0047, Japan*

[tanki.nobuyoshi@gmail.com](mailto:tanki.nobuyoshi@gmail.com)

**Tadao Kuwano, M. Sc.**

Graduate School of Health Sciences, Okayama University,

*2-5-1, Shikata-cyo, Kita-ku, Okayama-city, Okayama, 700-8558, Japan*

ta_kuwano2000@yahoo.co.j

**Kouichi Yabunaka, Ph. D.**

Graduate School of Medicine, the University of Tokyo,

*7-3-1 Hongo, Bunkyou-Ku, Tokyo, 113-0033, Japan*

kyabunaka-tky@umin.ac.jp
